# Human-Associated Methicillin-Resistant *Staphylococcus aureus* Clonal Complex 80 Isolated from Cattle and Aquatic Environments

**DOI:** 10.3390/antibiotics10091038

**Published:** 2021-08-25

**Authors:** Khuliso Ramaite, Mutshiene Deogratias Ekwanzala, John Barr Dewar, Maggy Ndombo Benteke Momba

**Affiliations:** 1 Department of Environmental, Water and Earth Sciences, Tshwane University of Technology, Arcadia Campus, Pretoria 0001, South Africa; 211148365@tut4life.ac.za (K.R.); ekwanzalamd@tut.ac.za (M.D.E.); 2 Centre for Antibiotic Resistance Research (CARe), University of Gothenburg, 41346 Gothenburg, Sweden; 3 Department of Infectious Diseases, Institute of Biomedicine, University of Gothenburg, 41346 Gothenburg, Sweden; 4 Department of Life and Consumer Sciences, University of South Africa, Florida Campus, Johannesburg 1709, South Africa; dewarj@unisa.ac.za

**Keywords:** husbandry soil, manure, water, livestock, methicillin-resistant *Staphylococcus aureus*

## Abstract

Background: Human-associated methicillin-resistant *Staphylococcus aureus* (HA-MRSA) has mainly been reported in South African pig and chicken farms. The prevalence of antibiotic-resistant genes (ARGs), virulence factors (VFs), and multilocus sequence types (MLSTs) associated with HA-MRSA in cattle farms has not been reported. Consequently, this study characterised LA-MRSA and its spread from cattle farms into the environment. Method: Husbandry soil (HS), nearby river water (NRW), animal manure (AM) and animal drinking water (ADW) were collected on and around a cattle farm. Presumptive MRSA isolates were identified from these samples using CHROMagar media and genotyped as MRSA sequence types (STs), selected ARGs, and VFs, using polymerase chain reaction. An MLST-based dendrogram was generated to link the farm MRSA strains with those in a nearby river. Results: The prevalence of MRSA was 30.61% for HS, 28.57% for ADW, 22.44% for NRW, and 10.20% for AM. Isolates from HS harboured the highest number of resistant genes, with 100% for *mecA*, 91.66% for *ermA*, and 58.33% for *blaZ*. However, no *ermC* or *tetM* genes were detected. MRSA isolates from AM harboured the lowest number of resistant genes. Only *sec* and *seq* enterotoxins were found in all the assessed MRSA isolates. MRSA from the farm revealed six STs (ST80, ST728, ST1931, ST2030, ST3247, and ST5440); all of STs belonged to clonal complex 80 (CC80). An MLST-based dendrogram based on the concatenated sequences of MLST genes under the maximum likelihood criterion revealed four clades of amalgamated MRSA isolates from various livestock environmental matrices, including the NRW. Conclusion: The results suggest that livestock environmental matrices might be reservoirs of MRSA that could subsequently disseminate through runoff to pollute water resources. Therefore, continued surveillance of HA-MRSA in livestock environments is warranted.

## 1. Introduction

Water is a basic requirement for humans and should be potable and free from pathogenic bacteria [1]. However, polluted rivers and lakes are critical drivers of the release, mixing, and persistence of antibiotic-resistant bacteria (ARB) and their antibiotic-resistance genes (ARGs), resulting in the exposure of humans to polluted surface water [2]. It has been estimated that 1.8 billion people, mainly in developing countries, consume unsafe water; this does not exclude South Africa [3]. Since it is a water-scarce country, many people living in this country, particularly those residing in rural areas and informal settlements, depend on untreated river water for multiple purposes, such as bathing and drinking [3]. According to Verlicchi and Grillini [4], South Africa’s population is estimated at 58.8 million. Based on statistical data gathered in 2018, 80.1% of the population lives in urban areas, 13.1% in traditional settlements (villages), and 5% live in informal housing or are informal settlers. Of these South Africans, 19%, around 3.8 million people, use potentially polluted untreated water or groundwater.

Environmental and clinical studies have reported a high ARB prevalence [5], including methicillin-resistant *Staphylococcus aureus* (MRSA). These bacteria are resistant to methicillin and almost all beta-lactamase (β-lactamase) antibiotics and represent a significant clinical concern in hospitals and communities [6,7]. As a result, MRSA is listed by the WHO as a high priority pathogen, as it is an ARB that causes major global disease outbreaks [8], including in South Africa [9]. Methicillin-resistant *Staphylococcus aureus* is divided into three epidemiological reservoirs, namely hospital-associated MRSA (HA-MRSA), community-associated MRSA (CA-MRSA), and livestock-associated MRSA (LA-MRSA) [10]. Since the first reports of MRSA in hospital settings, the rates of infection involving these organisms have increased rapidly [11]. Hospital-associated MRSA causes numerous nosocomial infections in both children and adults [11], among whom it is associated with treatment failure, leading to high morbidity and mortality [12]. MRSA prevalence in South African hospital settings is reported to be between 29% and 46% [13]. Two international multicentre studies have reported MRSA involvement in Staphylococcal infection in South Africa between 33% and 39%. A South African study conducted in public hospitals showed MRSA prevalence rates ranging between 30% and 60% [11]. Complementing this nosocomial increase in the presence of MRSA was a 31% increase in the prevalence of MRSA in human communities (CA-MRSA) [14], compromising effective antibiotic treatment [9].

Antibiotics are extensively used in agriculture, particularly in poultry and pig farming, followed by cattle farming [15]. These antibiotics are mainly used as growth promoters and for prophylaxis against diseases. In 2013, antibiotic use in livestock farming was estimated to be 131,109 tons per year, and this figure is projected to increase significantly by 2030 to exceed 200,000 tons per year [16]. However, the overuse and misuse of antibiotics has resulted in antibiotic resistance (AR) in bacteria expressing ARGs [17]. Such resistant bacteria and associated genes are excreted into the environment [18], and it is estimated that by 2050, AR will contribute to 10 million deaths each year [19]. Livestock-associated MRSA was first described in European pigs, and human infections resulted from zoonotic transmission [20]. In South Africa, a study conducted by Van Lochem et al. [21] estimated a 12% prevalence of MRSA in staphylococcal infections in large commercial piggeries. Furthermore, there is a link between ARGs circulating in the environment and those in clinical and farm settings [6]. Therefore, there is a need to investigate a possible route of transmission of MRSA via surface water by analysing MRSA and its ARGs in various livestock samples and nearby aquatic environmental samples.

The widespread use of antibiotics in agricultural practices has increased the dissemination of AR [22]. Antibiotics administered to livestock are poorly absorbed in the gut, and antibiotic residues are excreted in urine and faeces [23]. Because of the excessive use of antibiotics, excreted antibiotic residues in farms potentially lead to an increased number of resistant bacteria in the environment [23]. When resistant bacteria are excreted into the livestock environment, they disseminate to nearby settings, resulting in the introduction of resistant bacteria and genes into the human food chain. Humans can be exposed to such resistant bacteria, especially farmworkers and those who live near the farm [24]. Livestock farms are one of the primary sources of environmental ARGs present in different livestock wastes [18]. Antibiotic-resistant bacteria carrying ARGs are released into the livestock environment, such as soil, manure, and water [18]. Soil is a significant reservoir of AR in the environment [25]. In livestock waste, ARGs are frequently detected in the soil [26]. Many resistant genes in the soil are associated with the excessive use of antibiotics, agricultural practices, and manure [25]. Antibiotic-resistant genes can survive in soil for a prolonged period [18]. Up to 90% of the antibiotics used in animals are excreted into manure. Animal manure is a reservoir of AR associated with the emergence and dissemination of resistant bacteria that can potentially enter the environment [5,18,22]. Moreover, antibiotics in livestock are administered in animal feed or water. However, incidental spills or discharged antibiotics can be introduced into the environment [27]. Contamination of aquatic environments by animal manure and farm soil is a concern [28]. There are no reports of the presence of MRSA in husbandry soil, animal manure, animal drinking water, and nearby receiving water.

In South Africa, several multi-drug-resistant *S. aureus* and MRSA genes have been identified in clinical specimens and animal farms and aquatic environmental (drinking water) samples. These genes included *mecA*, *blaZ*, *aac (6′)-aph (2″)*, *ermC*, *tetK*, *vanA*, *vanB*, *tetM*, *aacA-aphD*, and *mecC* [6]. Antibiotic resistance genes originating from livestock farms has been reported to contaminate nearby receiving environments, posing a risk to humans using contaminated water [17,18]. In both humans and animals, *S. aureus* infection severity is based on the production of virulence factors encoding enterotoxins [29]. In a study by Amoako et al. [30], staphylococcal enterotoxins (*sea*, *sek*, *sep*, and *seq*) were identified in chicken carcasses. To better distinguish circulating strains, the evolutionary relationship between bacterial isolates was characterised by multilocus sequence typing (MLST) of seven housekeeping genes, where each gene sequence was determined to be a separate allele with each classified as the sequence type (ST) [31]. In South Africa, STs from cattle farms have neither been identified nor its subsequent dissemination from the farm to nearby aquatic environments investigated. Therefore, it is imperative to investigate circulating STs of MRSA and its associated ARGs in both livestock and nearby aquatic environments to provide information that can be used to monitor CA-MRSA.

Similarly, several studies have focused on poultry or pig farms. To our knowledge, no reports have described the presence and distribution of MRSA resistant genes, namely *mecA*, *ermA*, *ermC*, *tetM*, *blaZ*, and enterotoxins, *sea*, *seb*, *sec*, *see*, and *seq* in isolates from within and around a cattle farm. Research is also required to detail the STs circulating in a cattle farm setting and compare these to those present in an adjacent aquatic environment. This study aimed to characterise circulating MRSA in livestock farms by typing their ARGs, enterotoxins, and ST using MLST-based dendrogram analysis to reveal the MRSA sequence type isolated from various livestock sources and nearby aquatic environments.

## 2. Materials and Methods

Livestock associated with environmental samples (husbandry soil (HS), animal manure (AM), animal drinking water (ADW), and nearby river water (NRW)) were collected at the Tshwane University of Technology (TUT) research farm located in Honingnestkranz near Bon Accord, in Pretoria North. The farm is located in the City of Tshwane, Gauteng Province, South Africa. The TUT farm is 1172 m above sea level (latitude 25°37′ S, longitude 28°16′ E). The farm has a few existing buildings, including livestock pens and agricultural infrastructure. It mainly breeds cattle for commercial meat, and the cattle ranch comprises soil, AM, and water troughs from which animals drink water. The nearby water was collected from a river that receives water from the Bon Accord dam. Informal settlers use nearby river water for household purposes, such as bathing and drinking. Eight samples were collected from the cattle farm on 24 occasions between October and December 2018 to provide 192 environmental samples. Solid samples consisted of duplicate 300-g samples of solid HS and AM, collected aseptically in sterile propylene bottles using sterile spatulas. To ensure local sample homogeneity, two true replicates consisting of a mixture of five pseudo-replicates were taken within a 1 m^2^ quadrat around the farm to generate reliable and representative results, as described by Ekwanzala et al. [32]. In addition, duplicates of 1000-mL water samples were collected in sterile bottles using a telescoping pole. Water collection involved sampling ADW provided to farm livestock and water from a nearby stream (NRW), located 0.1 km away from the farm, used for recreational bathing and drinking by people living near the farm and downstream from the farm. Samples were transported to the laboratory on ice at 4 °C and processed within 4 h of collection.

### 2.1. Preparation of Solid Samples

Solid samples were preoared using the water-displacement method previously described by Abia et al. [33], with some modifications. Briefly, each type of solid sample (HS or AM) of approximately 300 g was gradually and aseptically transferred into a graduated 1-L sterile Durham bottle containing 400 mL of 1× phosphate-buffered saline (PBS), 137 mM NaCl, 2.7 mM KCl, 8 mM Na_2_HPO_4_, and 2 mM KH_2_ until the 500 mL mark was reached. The bottle was then hand-shaken vigorously for about two minutes to detach the bacteria from the soil or manure to disperse them through the liquid. Thereafter, it was allowed to stand for approximately 30 min for the solid particles to settle. The supernatant (100 mL) was then extracted to ascertain the prevalence and genetic characteristics of the MRSA strains. No processing was performed for ADW and NRW. As described below, 100 mL of each water sample was analysed for MRSA isolates prior to genetic analysis.

### 2.2. Isolation and Identification of MRSA

For the isolation of MRSA, enrichment cultures were prepared by adding 100 mL of each sample (HS, AM, ADW, or NRW) to separate 200-mL volumes of tryptone soy broth (TSB, OXOID) (Thermo Scientific, Johannbesburg, South Africa). Broths were incubated overnight at 37 °C. Thereafter, a loopful of each enriched culture was streaked onto their respective CHROMagar™ Staph aureus agar (Media mage, Johannesburg, South Africa) and CHROMagar™ MRSA agar (Media mage, South Africa), to isolate *Staphylococcus aureus* and MRSA, respectively. A methicillin-resistant positive control, namely *S. aureus* subsp. *aureus* (ATCC^®^ 43300™), and negative control, *Escherichia coli* (NCTC^®^ 11954™) (Thermo Scientific, Johannbesburg, South Africa), were used as the reference strains. Typical colonies that grew on CHROMagar™ *S. aureus* agar and CHROMagar™ MRSA agar were confirmed by a positive catalase reaction using 3% hydrogen peroxide.

Of the 180 isolated MRSA isolates, 100 randomly selected colonies that showed positive catalase test results, 25 isolates from each matrix, were further confirmed by matrix-assisted laser desorption ionisation time-of-flight mass spectroscopy (MALDI-TOF-MS analysis) at the Department of Microbiology and Plant Pathology, University of Pretoria (MALDI-TOF Diagnostic Service, Pretoria, South Africa). Briefly, to confirm the catalase-positive colony’ identity, each colony was prepared and transferred onto a MALDI Biotarget 48 sample spot according to the manufacturer’s protocol (Bruker, Instruction for use MALDI BIOTARGET 48, Middlesex County, MA, USA). After the spot had dried, 1 µL of HCCA matrix solution was added to each spot, and the spot was allowed to air dry before being loaded onto the mass spectrometer (Sciex, Concord, ON, Canada). Data were obtained from the MALDI-TOF machine using the MBT Explorer Software plus MBT Compass Library.

### 2.3. DNA Extraction

Following the results of the MALDI-TOF analysis, confirmed MRSA sample isolates were thawed for DNA extraction. DNA was extracted from preserved cultures of MRSA using the ZR Fungal/Bacterial DNAMiniPrep™ kit following the manufacturer’s instructions (Inqaba Biotechnical Industries, Pretoria, South Africa). The quantity and quality of the isolated gDNA were determined using a NanoDrop™ 2000 spectrophotometer (Thermo Scientific, Johannbesburg, South Africa). The extracted gDNA suspension was stored at −20 °C until ready for polymerase chain reaction (PCR) to detect ARGs, enterotoxins, and MLST.

### 2.4. Detection of ARGs from Isolated MRSA Strains

Methicillin-resistant *S. aureus* isolates were further analysed using conventional PCR amplification to detect *mecA*, *ermA*, *ermC*, *tetM*, and *blaZ*. Each PCR was performed in a total volume of 25 µL containing 5 µL of template DNA, 0.5 µL of forward primer (10 µM) and 0.5 µL of reverse primer (10 µM), 12.5 µL of *Taq 2X* master mix, and 6.5 µL of nuclease-free water (Inqaba Biotechnical Industries, Pretoria, South Africa). Amplification was carried out in a MiniAmp Plus thermal cycler (ThermoFisher, Johannbesburg, South Africa). The following cycling conditions were used. The heating lid was set at 110 °C, and an initial denaturation at 95 °C for 30 s. This was followed by 30 cycles of denaturation at 95 °C for 30 s annealing at 55 °C for 1 min, extension at 68 °C for 1 min, and cycling was followed by a final extension step at 68 °C for 5 min. The expected band sizes of the PCR products were visualised by electrophoresis on a 1% agarose gel prepared in 1 × TAE buffer and stained with ethidium bromide (0.5 µg/mL). Electrophoresis was conducted for 60 min at 100 V, and the amplicons in the gel were visualised under ultraviolet light using a Syngene Gel documentation system (Vacutec, Roodepoort, South Africa). The primer sequences are shown in Table 1.

### 2.5. Enterotoxins Detection in MRSA Isolates

The presence of staphylococcal enterotoxins a (*sea*), b (*seb)*, c (*sec*), e (*see*), and q (*seq*) genes was assessed using multiplex PCR (mPCR) using the primers described in Table 1. Each mPCR was conducted in a total volume of 25 µL. The fluid consisted of 6 µL of template DNA, 0.5 µL of each (*sea*, *seb*, *sec*, *see*, and *seq*) forward primer (10 µM) and 0.5 µL of each (*sea*, *seb*, *sec*, *see*, and *seq*) reverse primer (10 µM), 12.5 µL of *Taq* 2× master mix, and 1.5 µL of nuclease-free water (Inqaba Biotechnical Industries, Pretoria, South Africa). Amplification was conducted as described in the previous section to amplify and detect resistance genes in MRSA strains. Likewise, agarose gel electrophoresis of PCR amplicons and their visualisation were performed as described in the above section.

### 2.6. Multilocus Typing of MRSA Isolates

Using multilocus sequence typing, we characterised seven housekeeping genes, ca. carbamate kinase (*arcC*), shikimate dehydrogenase (*aroE*), glycerol kinase (*glpF*), guanylate kinase (*gmk*), phosphate acetyltransferase (*pta*), triosephosphate isomerase (*tpi*), and acetyl coenzyme A acetyltransferase (*yqiL*), was performed according to the method described by Enright et al. [31]. In the current study, MLST fragments were amplified using the primers listed in Table 1. The amplification was carried out in a 25-µL reaction volume using the same reaction components and cycling conditions described in the previous section to identify the MRSA’s ARGs. Amplicons were visualised using electrophoresis on an ethidium bromide-stained agarose gel as described above. 

Sequencing was performed at Inqaba Biotech (Pretoria, South Africa). The dideoxy Sanger sequencing in the forward direction was used with the primer sets listed in Table 1. For this procedure, the Big Dye™ Terminator Cycle Sequencing Kit for ABI3130XL was used according to the manufacturer’s instructions, and the gel was run on a 3130XL sequencer (NimaGen B.V., Nijmegen, The Netherlands).

### 2.7. Bioinformatic Analysis

Sequences were edited using MegaX and queried using the BLASTn algorithm (https://blast.ncbi.nlm.nih.gov/Blast.cgi, accessed on 21 September 2020) in the National Center Biotechnology Information to confirm the *S. aureus* homology sequences. Briefly, all seven housekeeping genes were typed (*arcC*, *aroE*, *glp*, *gmk*, *pta*, *tpi*, and *yqiL*) and concatenated using MEGA X [37]. The MUSCLE algorithm was used to align the concatenated sequences [38]. The evolutionary history was inferred using the maximum likelihood method and the Tamura–Nei model [39]. The tree was drawn to scale, with branch lengths measured as the number of substitutions per site. This analysis involved 33 nucleotide sequences. Codon positions included were 1st + 2nd + 3rd + Noncoding. Evolutionary analyses were conducted using MEGA X [37]. The inferred MLST-based dendrogram was annotated using iTol [40], where STs, ARGs, and enterotoxins were allocated to their respective strains.

To understand the relatedness between our isolated MRSA and those isolated from the clinical settings, we constructed a phylogenetic tree using MLST sequences of our MRSA isolates against ST sequences downloaded from the PubMLST database (https://pubmlst.org/saureus/, accessed on 2 October 2020) located in South Africa and those identified in published articles from South Africa [14,41,42,43]. All isolated sequences mentioned above were aligned and analysed as described above. *Staphylococcus epidermis* was used as an outgroup.

### 2.8. Statistical Analysis

The prevalence of *S. aureus* and MRSA, ARG distribution, and enterotoxins were plotted using Microsoft Excel PowerPoint^®^ 2016 (Microsoft Corporation, Redmond, WA USA). The prevalence of positive samples for each matrix was expressed as the percentage of positive samples from the total number of samples tested. Fischer’s exact test was used to evaluate the difference in MRSA prevalence among the four matrices. Theanalysis was performed at the 95% confidence limit (α = 0.05).

## 3. Results

**Figure 1:**
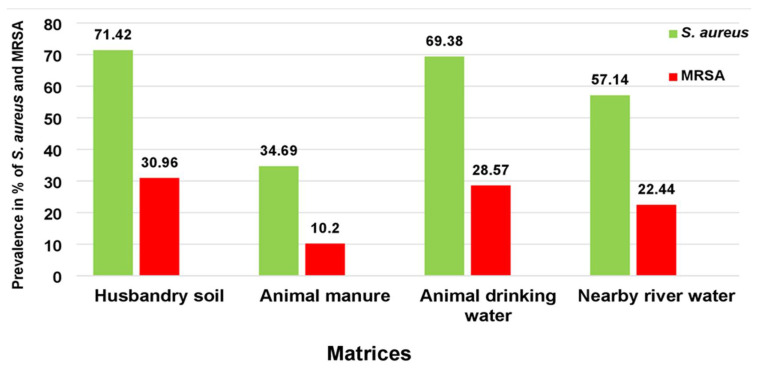
The prevalence of culture-positive samples for *S. aureus* compared to the MRSA culture-positive samples per sample matrix.

From October to December 2018, 192 samples were collected, comprising 48 samples from each matrix (HS, AM, ADW, and NRW). Of the 192 samples, 59.37% (114/192) were presumptively positive for *S. aureus* according to growth on CHROMagar™ Staph aureus medium. The prevalence of presumptive colonies using CHROMagar™ MRSA was 23.43% (*n* = 45). Of the presumptive *S. aureus* isolates (*n* = 114), the prevalence was high in HS at 72.91% (*n* = 35) and in ADW at 70.83% (*n* = 34), followed by NRW at 58.33% (*n* = 28), while AM showed the lowest presumptive prevalence of *S. aureus* at 35.1% (*n* = 17). For presumptive MRSA isolates (*n* = 45), the highest prevalence was found in HS at 31.25% (*n* = 15), followed by ADW at 29.16% (*n* = 14) and NRW at 22.91% (*n* = 11). The lowest prevalence was noted in AM samples at 10.41% (*n* = 5). Figure 1 represents the prevalence of culture-positive samples for *S. aureus* compared to the positive samples for MRSA per matrix for a simplified interpretation of the data.

The catalase test confirmed *S. aureus* and MRSA, and MRSA isolates were further confirmed by MALDI TOF Biotyper analysis. Of the randomly selected 100 presumptive isolates from all matrices, 33 isolates were positive for MRSA. These comprised MRSA isolates from HS (*n* = 12; 12%) and from ADW (*n* = 12; 12%), followed by isolates from NRW (*n* = 6; 6%) and isolates from AM (*n* = 3; 3%). Other bacteria accounted for the remaining 67% of isolates, which were identified as *Lysinibacillus boronitolerans* (20%; *n* = 20), *Lysinibacillus fusiformis* (10%; *n* = 10), and *Bacillus cereus* (7%; *n* = 7). The remaining 30 isolates were not identified in this study. 

### 3.1. ARGs Typed in MRSA Isolates

**Figure 2:**
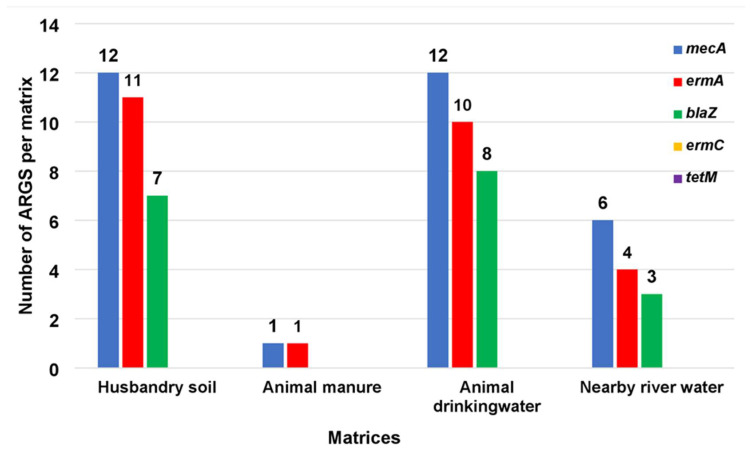
The distribution of five selected ARGs assessed in isolated MRSA from sampled matrices.

Of the five selected ARGs (methicillin (*mecA*), macrolides (*ermA* and *ermC*), tetracycline (*tetM*), and beta-lactamase (*blaZ*)), analysed in the MRSA cultures (*n* = 33), the most commonly detected gene was *mecA* (93.93%; *n* = 31), followed by the *ermA* gene (78.78%; *n* = 26) and *blaZ* (54.54%; *n* = 18). No *ermC* and *tetM* genes were detected in any of the isolates. Regarding the detection of these genes in HS isolates, the *mecA* gene was amplified in all 12 HS isolates, the *ermA* gene in 11 isolates, and the *blaZ* gene in seven isolates. Of the ADW isolates, all 12 contained the *mecA* gene, with 10 isolates containing the *ermA* gene and eight isolates containing the *blaZ* gene. For the NRW isolates (*n* = 6), *mecA* was detected in all six isolates, with *ermA* detected in four isolates and *blaZ* detected in three isolates. In three AM isolates, the *mecA* and *ermA* genes were detected but only one isolate was detected. The distribution of the selected ARGs assessed in this study is illustrated in Figure 2.

### 3.2. Enterotoxins Detected in Isolated MRSA

The prevalence of enterotoxins was determined in all 33 MRSA isolates. These were screened for the presence of five enterotoxins (*sea*, *seb*, *sec*, *see*, and *seq*), but only the *sec* and *seq* genes were detected in all the matrices (HS AM, ADW, and NRW).

### 3.3. Sequence Types and MLST-Based Dendrogram

**Figure 3:**
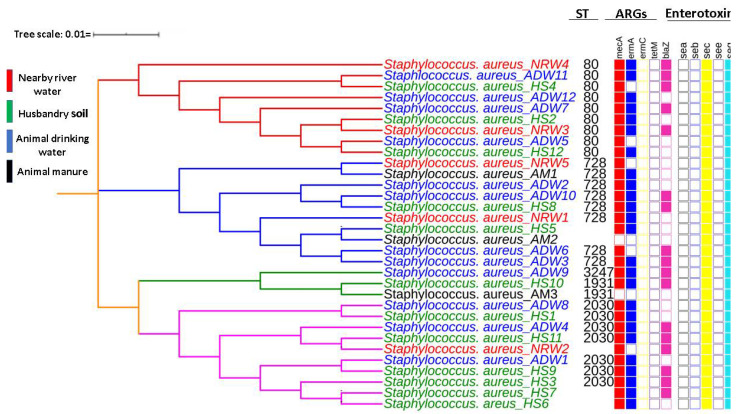
MLST-based dendrogram based on the concatenated of the seven housekeeping genes of MRSA isolates from HS, AM, ADW, and NRW highlighting selected ARGs and enterotoxins.

Of the potential sequence types, nine MRSA STs were typed as ST80, followed by eight and seven MRSA belonging to ST728 and ST2030, respectively. Four isolates were classified as ST1931 and ST3247. Another five isolates could not produce ST, as one or two housekeeping genes contained gaps. The inferred MLST-based dendrogram based on the concatenation of the seven housekeeping genes is shown in Figure 3.

The tree with the highest log likelihood (−11126.19) is shown. Initial trees for the heuristic search were obtained automatically by applying the Neighbour-Join and BioNJ algorithms to a matrix of pairwise distances estimated using the maximum composite likelihood approach and then selecting the topology with a superior log-likelihood value. The final dataset contained 4275 positions. The MLST-based dendrogram of the concatenated sequences generated using the maximum likelihood method and the Tamura–Nei model revealed three distinct lineages in the MRSA isolate strains, even though most typed STs belonged to clonal complex 80 (CC80). Considering these results, all three clades represent amalgamated strains from HS, AM, ADW, and NRW. The first clade in red consisted of ST80 representing the evolutionary relationships among a mixture of isolates from HS, ADW, and NRW except for AM isolates. The second clade in blue, consisting mainly of ST728 and two untypable STs, showed a mixture of isolates from all matrices. The third clade represented two-sub clades, the mixture of isolates from all matrices consisting of ST3247, 5440, 1931, and 2030. After sequence analysis, we observed that all STs had only one single nucleotide polymorphism in the tpi gene. No SNPs were observed among isolates of the same clade.

**Figure 4:**
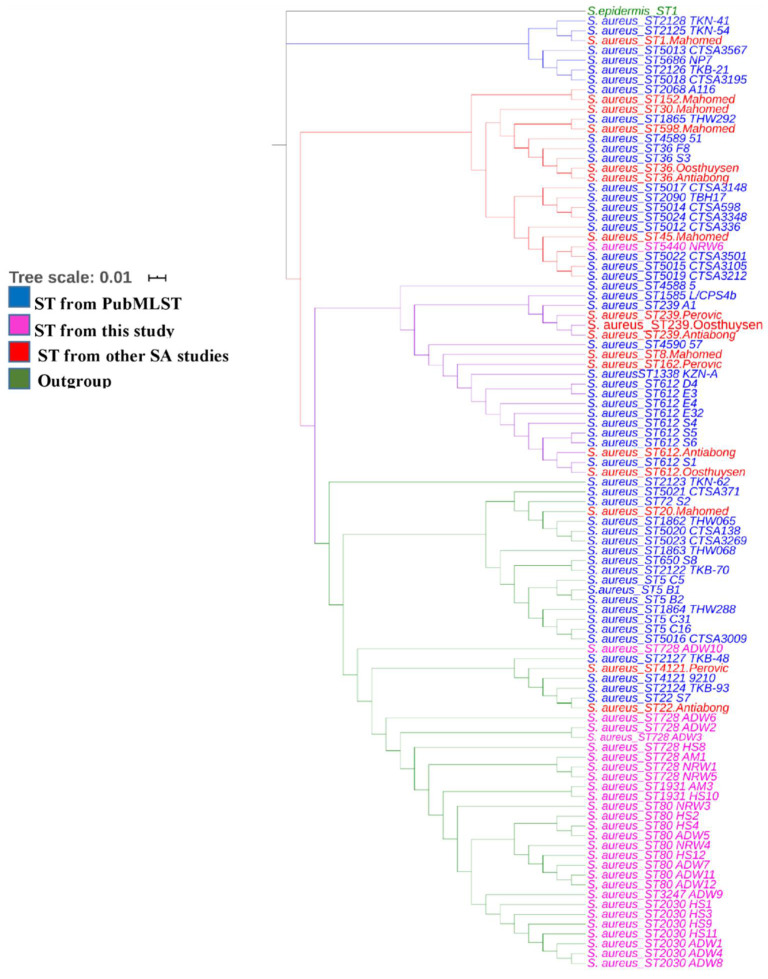
The phylogenetic tree of *S. aureus* isolates generated involving our isolates and those from other South African studies using iTol v.3.The isolates from the PubMLST database are coloured blue, ST for this study are pink, other South African studies are red, and the outgroup is in green.

The phylogenetic tree of our isolates, those from other South African clinical studies (Perovic et al., 2006; Oosthuysen et al., 2014; Antiabong et al., 2017; Mahomed et al., 2012) and those from PubMLST revealed intermixed *S. aureus* isolates from the South African database. Other isolates from MRSA articles in South Africa and our current study clustered based on their sequence type, as shown in Figure 4. Both isolates from the South African database and other MRSA articles clustered together in different clades. Four isolates in our current study showed intermixed MRSA isolates (ST80, ST1931, ST728, and ST2030) from various matrices, namely HS, AM, and NRW, but clustered together under one clade based on ST. However, ST728 (WF10) isolates did not cluster with other isolates.

## 4. Discussion

Globally, infection with methicillin-resistant *S. aureus* and its most recent clone, vancomycin-resistant MRSA, is of concern in hospitals and communities [44]. A South African hospital study suggested an increase in CA-MRSA, contributing to high mortality [45]. A possible contributor to CA-MRSA may involve the use of antibiotics in livestock management to promote cattle growth and prevent or treat diseases in cattle. Such antibiotics are partially metabolised in the cattle gut, and once excreted, they can spread into the environment to potentially contaminate manure, soil, and water [27]. Therefore, it is essential to investigate ARGs in livestock, the associated environment, and their subsequent distribution into aquatic environments used by humans for drinking purposes. The current study characterised circulating MRSA in livestock farms by typing their STs, resistant genes, and enterotoxins using MLST-based dendrogram analysis to reveal the MRSA sequence type isolated from various livestock sources. Few studies have characterised MRSA resistant genes and enterotoxins from different livestock-associated environments. Hence, this study described MRSA ARGs and enterotoxins from various livestock-associated environmental matrices, potentially a source of dissemination into the environment.

In the current study, the prevalence of *S. aureus* in HS was 72.91%, while the prevalence of MRSA in HS was lower at 31.25%. The prevalence of *S. aureus* is comparable to the overall prevalence of *S. aureus* as assessed in the study by Dweba et al. [46]. Although soil is not a natural habitat for *Staphylococcus aureus*, a high prevalence of MRSA in soil has been reported in livestock-associated soil [47]. The relatively high prevalence of *S. aureus* in soil may act as a contamination depot, allowing subsequent AM and NRW with these bacteria. Moreover, soil is considered an AR environmental reservoir [25]. Although manure is regarded as a hot spot for pathogens that harbour resistant genes [48], there are no reports of *S. aureus* and MRSA reports in AM from a livestock environment. Thus, the current study reports the first-time prevalence figures in livestock manure of 35.25% and 10.41% for *S. aureus* and MRSA, respectively. Compared to the prevalence of these bacteria in HS, relatively few *S. aureus* or MRSA isolates were obtained from manure samples. An explanation for this result could be that the survival of ARB in manure differs, depending on the bacterial pathogen and the environment in which they are excreted [49]. The results showed that the prevalence of *S. aureus* in ADW was high at 70.83%, a rate that is almost comparable with the reported prevalence of *S. aureus* in ADW (58.33% and 60%) on two South African farms [46]. Prior to the present study, the prevalence of *S. aureus* and MRSA in South African river water in the vicinity of livestock farms was unknown. Therefore, this study reports for the first time prevalence figures of 58.33% and 22.91% for *S. aureus* and MRSA, respectively, in river water. Although the prevalence of MRSA has not previously been reported in HS and ADW, this study revealed a prevalence of 31.25% and 29.16% in these matrices, respectively.

The distribution of ARGs for MRSA has been reported in a few studies in relation to recreational water, farm soil, and water from rivers that receive water from domestic and hospital wastewater [50]. The current study detected ARGs in MRSA that can contribute to treatment failure in clinical settings to treat MRSA infections. This study characterised MRSA from a livestock farm by detecting ARGs from the HS, AM, ADW, and NRW sampling sites, and except for isolates from AM, a similar ARG profile was determined in these three matrices. Compared to the other three sampling sites, the current study showed that HS preferred to sustain MRSA isolates containing the most significant proportion of ARGs. This was indicated by the fact that all the MRSA isolates in HS (*n* = 12) had the *mecA* gene (100%; *n* = 12), with most (91.66%; *n* = 11) containing the *ermA* gene, while more than half (58.33%; *n* = 7) had the *blaZ* gene. *Ermc* and *tetM* genes were not detected. Variation in the ARG profile might be due to varying bacterial profiles in different soils from various regions and differential ARB enrichment in soil bacteria selected according to available contaminants, such as antibiotic residues within a particular soil [51].

In contrast to the relative abundance of MRSA isolates and ARGs in HS, the results of the current study indicated low detection of both MRSA isolates and ARGs in cattle manure (AM) samples. Thus, only one isolate (*n* = 1) of the total isolates (*n* = 3) was noted, and it harboured only the *mecA* gene (*n* = 1) and the *ermA* gene (*n* = 1) but not the *ermC*, *tetM*, or *blaZ* genes. This observation may be explained by antibiotics, such as β-lactamase and macrolides, which degrade rapidly within a few days. However, most of these compounds are transferred from manure to the soil [52]. Consequently, the current study indicated a similar profile of MRSA and ARGs in ADW as that found in HS; therefore, the *mecA* gene was detected in all ADW isolates (100%; *n* = 12), with most (83.33%; *n* = 10) containing the *erm A* gene and over half (66.66%; *n* = 8) carrying the *blaZ* gene. No *ermC* or *tetM* genes were detected. Apart from the fact that the current study did not detect the *tetM* gene in isolates, the detection of ARGs was comparable to that reported in a study involving a cattle farm in the Eastern Cape Province of South Africa [53]. Aquatic environments act as a transmission route for the spread of AR to communities, *mecA* was detected in all six NRW isolates, while *ermA* was detected in 66.66% (*n* = 4) of isolates and *blaZ* was detected in half (50%; *n* = 3) of the isolates. These findings are similar to those described by Akanbi et al. [54] who reported the presence of *mecA, blaZ*, and *ermA*.

Virulence factors in MRSA are responsible for bacterial colonisation and pathogenicity, triggering disease [55]. *Staphylococcus aureus* produces extracellular staphylococcal enterotoxins. They are classified as *sea*, *seb*, *sec*, *sed*, *see*, and *seg*. The 12 HS isolates all showed only two enterotoxins, *sec* and *seq*. Likewise, only these two enterotoxins were detected in all AM, AW, and NRW isolates. No *sea*, *seb*, or *see* enterotoxins were detected. This result contrasts with those reported by Ramessar and Olaniran [50], who detected the *sea* gene in more than half of the screened river isolates (57.50%). The consistent detection of *sec* and *seq* genes in all MRSA isolates from all the matrices selected in the current study suggests similar origins of the LA-MRSA isolates and the proximity of these gene sequences to the genome of MRSA strains. From an epidemiological perspective, the LA-MRSA resistance profiles can cause infections, particularly the microbiome in HS and NRW.

The MLST results indicated that all revealed STs (80, 728, 1931, 2030, 3247, and 5440) belonged to the complex clone (CC) 80. This result suggests that this research type should be expanded to include more samples to reveal a better spatial and temporal picture of STs and the environmental mobility and potential variation in *Staphylococcus* strains. The STs within CC80 share the following five common alleles: *arc* (*n* = 1), *aroE* (*n* = 3), *glp* (*n* = 1), *gmk* (*n* = 14), and *pta* (*n* = 11). Several studies have reported widespread MRSA-ST80 in 38 countries in community and hospital settings [56]. Moreover, the results showed that the most commonly detected ST was ST80 (CC80-MRSA-IV), occurring in eight isolates. These included isolates from HS (*n* = 3), ADW (*n* = 3) and NRW (*n* = 2). This ST was followed by ST728, with seven occurrences in all assessed matrices in terms of the detection rate. Molecular characterisation of MRSA revealed that isolates from HS, ADW, and NRW were clustered in all clades, indicating that aquatic environments may be contaminated with ARGs of MRSA harbouring enterotoxins. The results of this study revealed that CC80 from various matrices differs from the endemic clone ST612-CC8-t1257-SCCmec_IVd(2B) isolated from a poultry food chain in KwaZulu-Natal. This result suggests the possibility that in Gauteng Province, the circulating strain in livestock farms might be ST80 (CC80-MRSA-IV), a sequence type similar to the ST CC80-MRSA-IV that circulates as a CA-MRSA in the Middle East [57]. A study pointed out that the prevalence of a sequence type is determined by geographical area [58]. This result further suggests the need to expand such research to monitor such clones more closely to control CA-MRSA endemic CC80 infection. To the best of our knowledge, this is the first study to identify ST80(CC80-MRSA-IV) in South Africa. Sequence isolates from the South African database clustered in different clades based on their ST, in the first clade (blue) from different sources, such as nasal swabs, blood, other unknown sources, and one isolate from the clinical setting. The second clade (red) showed an intermix of isolates clustered together from different sources retrieved from the database from various sources and other articles. Interestingly, an isolate from our study ST5440 (NW6) clustered in one clade with isolates from the clinical setting and the South African database. The second isolate in our study, ST728 (WF10), also clustered with isolates from the clinical setting and South African database, both showing close relatedness of our isolates to those from the clinical settings and those that have previously been identified in South Africa.

This study had limitations. These results are representative of cattle and aquatic environments. Owing to constraints of time and cost, the target of all positive samples was not achieved. However, molecular techniques were used to determine selected ARGs, namely *mecA*, *ermA*, *ermC*, *tetM*, and *blaZ* and enterotoxins of MRSA isolates, which are clinically relevant. Whole-genome sequencing will be considered in future studies. Despite these limitations, our findings suggest that livestock environmental matrices might be reservoirs of MRSA that could subsequently disseminate through runoff to pollute water resources. Therefore, continued surveillance of CA- or LA-MRSA from the livestock environment is warranted.

## 5. Conclusions

This study investigated the genetic characteristics of MRSA present in cattle farm environmental matrices (HS, AM, and ADW) to track their spread into an environmental matrix, such as NRW. The study highlighted integrated genes, such as ARGs and enterotoxins, which confer survival advantages, particularly MRSA strains. The study showed the highest prevalence of MRSA (30.61%) in the HS livestock environmental matrix, whereas the AM matrix showed the lowest prevalence of MRSA (10.20%). These results suggest that farm soil and the water supplied to livestock are essential MRSA reservoirs in a cattle farm environment. The data on ARGs in AM are limited. It is tempting to speculate that the similarity in ARG profiles between the soil, ADW, and the nearby river water links the MRSA isolates and their ARG profiles from these three matrices. MRSA isolates are rapidly lost from AM to the soil, where they may eventually disseminate to NRW. The combination of ARGs and enterotoxins found in LA-MRSA that can spread from livestock into the water environment warns of the dangers of potentially widespread dissemination of ARGs and virulent bacterial strains. Multilocus sequence typing results allowed the detection of all LA-MRSA STs (ST80, ST728, ST1931, ST2030, ST3247, and ST5440) belonging to CC80. Epidemiological data showed that CC80 could be identified in animal farms; however, to our knowledge, no study has previously identified CC80 in cattle farms. This result implies that the reported CC80 clones from CA-MRSA worldwide might have originated from livestock environments. The inferred MLST-based dendrogram of MRSA showed intermixed clades of MRSA isolated from different environments. Therefore, this study recommends the implementation of effective water quality control measures. Health training needs to be provided to individuals living close to rivers that regularly use river water to prevent outbreaks in the community due to antibiotic-resistant bacteria, especially CA-MRSA.

## Figures and Tables

**Table 1:** Antibiotic-resistance, virulence, and housekeeping gene primers used in this study.

Gene Abbreviation	Primer Sequence (F: Forward, R: Reverse) 5′ to 3′	Product Size (bp)	Annealing Temp (°C)	References
**Antibiotics resistance genes**				
*mecA*	F-AAAATCGATGGTAAAGGTTGGC R-AGTTCTGCAGTACCGGATTTGC	532	55	[34]
*ermA*	F-AAGCGGTAAACCCCTCTGA R-TTCGCAAATCCCTTCTCAAC	190	55	
*ermC*	F-AATCGTCAATTCCTGCATGT R-TAATCGTGGAATACGGGTTTG	299	55	
*tetM*	F-AGTGGAGCGATTACAGAA RCATATGTCCTGGCGTGTCTA	158	55	
*blaZ*	F-ACTTCAACACCTGCTGCTTTC R-TGACCACTTTTATCAGCAACC	173	55	[35]
**Enterotoxins**				
*sea*	F-TTGCGAAAAAAGTCTGAA TTGC R-ATTAACCGAAGGTTCTGTAGAAGTA	552	55	[36]
*seb*	F-TCGCATCAAACTGACAAACG R-AGGTACTCTATAAGTGCCTGCCT	477	55	
*sec*	F-CTCAAGAACTAGACATAAAAGCTAGG RTTATATCAAAATCGGATTAACATTATC	271	55	
*see*	F-AGGTTTTTTCACAGGTCATCC R-CTTTTTTTTCTTCGGTCAATC	178	55	
*seq*	F-AATCTCTGGGTCAATGGTAAGC R-TTGTATTCGTTTTGTAGGTATTTTCG	122	55	
**Housekeeping genes**				
*arcC*	F-TTGATTCACCAGCGCGTATTGTC R-AGGTATCTGCTTCAATCAGCG	456	55	[31]
*aroE*	F-ATCGGAAATCCTATTTCACATTC R-GGTGTTGTATTAATAACGATATC	456	55	
*glpF*	F-CTAGGAACTGCAATCTTAATCC R-TGGTAAAATCGCATGTCCAATTC	465	55	
*gmk*	F-ATCGTTTTATCGGGACCATC R-TCATTAACTACAACGTAATCGTA	429	55	
*pta*	F-GTTAAAATCGTATTACCTGAAGG R-GACCCTTTTGTTGAAAAGCTTAA	474	55	
*tpi*	F-TCGTTCATTCTGAACGTCGTGAA R-TTTGCACCTTCTAACAATTGTAC	402	55	
*yqiL*	F-CAGCATACAGGACACCTATTGGC R-CGTTGAGGAATCGATACTGGAAC	516	55	

## Data Availability

Not applicable.
